# Monomorphic ventricular tachycardia as the primary presentation of an anterior STEMI

**DOI:** 10.1002/ccr3.2324

**Published:** 2019-07-26

**Authors:** Jens Aamann Andersen, Phillip Freeman, Jacob Mosgaard Larsen, Niels Holmark Andersen

**Affiliations:** ^1^ Department of Cardiology Aalborg University Hospital Aalborg Denmark

**Keywords:** ischemia, magnetic resonance imaging, ST‐elevation myocardial infarction, ventricular tachycardia

## Abstract

Traditionally sustained monomorphic ventricular tachycardia (SMVT) is associated with areas of myocardial scar such as that of chronic coronary artery disease. We present a case of SMVT in the initial setting of an acute myocardial infarction in a previously healthy individual suggesting that acute ischemia can give rise to SMVT.

## 1 INTRODUCTION

Sustained monomorphic ventricular tachycardia (SMVT) is a well‐described complication in coronary artery disease (CAD) but is an uncommon initial finding in the setting of acute myocardial infarction (AMI). We present a case of SMVT as the initial presentation of an anterior ST‐elevation myocardial infarction (STEMI) in a previously healthy individual.

## 2 CASE REPORT

**Figure 1:**
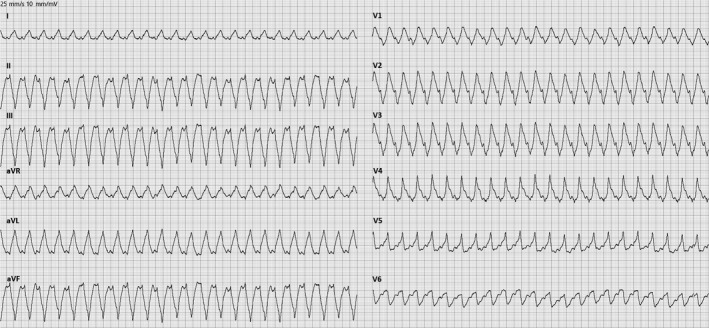
ECG at first medical contact showing sustained monomorphic ventricular tachycardia

During a recreational orienteering race, a 69‐year‐old man with no previous history of heart disease developed sudden chest pain and malaise. As symptoms persisted, emergency medical services were called, and an ambulance dispatched. Within 90 minutes of the debut of symptoms, paramedics arrived. At first medical contact, the patient had ongoing chest pain but did not report palpitations or syncope. The patient denied having experienced cardiac symptoms before, did not have a family history of cardiovascular disease and was a never‐smoker without a history of excessive alcohol consumption. Vital signs were stable apart from tachycardia at a rate of 157 beats per minute. A 12‐lead ECG was recorded demonstrating SMVT (Figure 1). The patient was treated with 300 mg aspirin, 0.4 mg glyceryl nitrate, and 70 µg fentanyl at the site. As the patient was awake and assessed hemodynamically stable, prehospital cardioversion was not performed.

**Figure 2:**
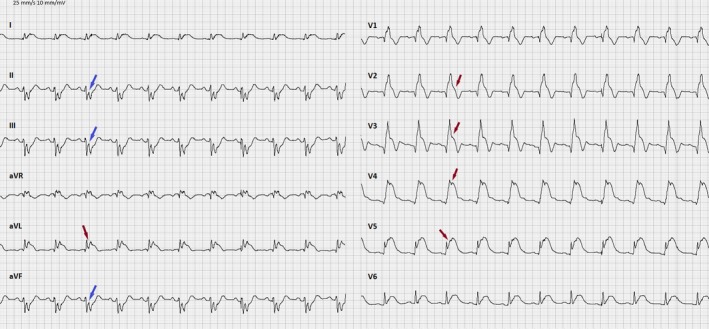
ECG after cardioversion showing anterior ST elevation (red arrows) and reciprocal ST depression (blue arrows)

**Figure 3:**
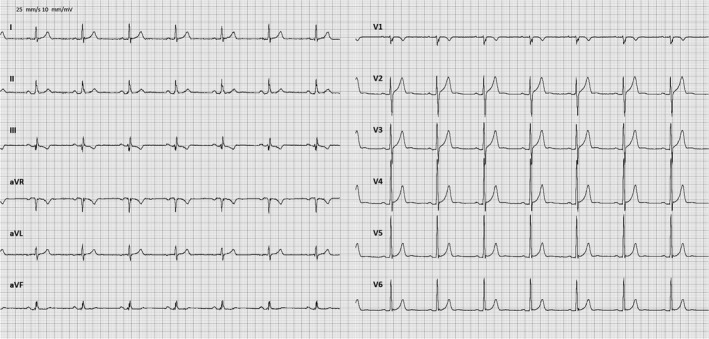
ECG from 2 years prior to index event

**Figure 4:**
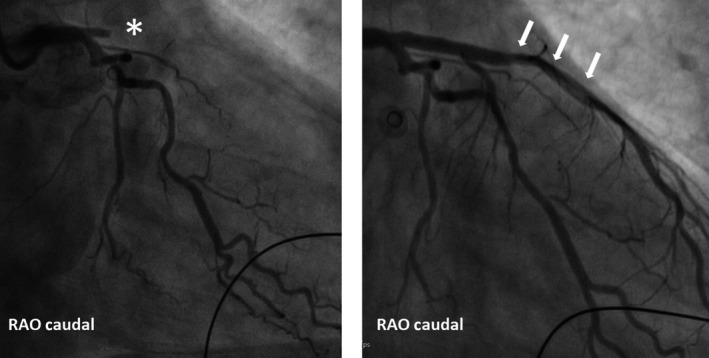
Left hand panel shows an acute coronary angiogram with a proximal LAD occlusion (*). The right hand panel shows the LAD after PCI (white arrows)

Upon admission to hospital the SMVT persisted and as the patient was now hypotensive with a blood pressure of 92/69 mm Hg acute cardioversion using a single 200‐joule biphasic shock was performed. After the restoration of sinus rhythm, the chest pain persisted, and a new 12‐lead ECG was obtained revealing pronounced anterior ST elevation and a right bundle branch block (Figure 2) that was not present on a ECG from the patient recorded 2 years prior (Figure 3). Urgent cardiac catheterization was performed demonstrating single vessel disease in a right dominant system with an acute proximal left anterior descending (LAD) occlusion that were treated with primary percutaneous coronary intervention (PPCI) (Figure 4).

Following successful revascularization, SMVT recurred and 300 mg amiodarone was administered intravenously. Since the arrhythmia persisted and as the patient experienced loss of consciousness, cardiopulmonary resuscitation with DC‐cardioversion was initiated resulting in termination of the SMVT with a return of spontaneous circulation time of <1 minute.

**Figure 5:**
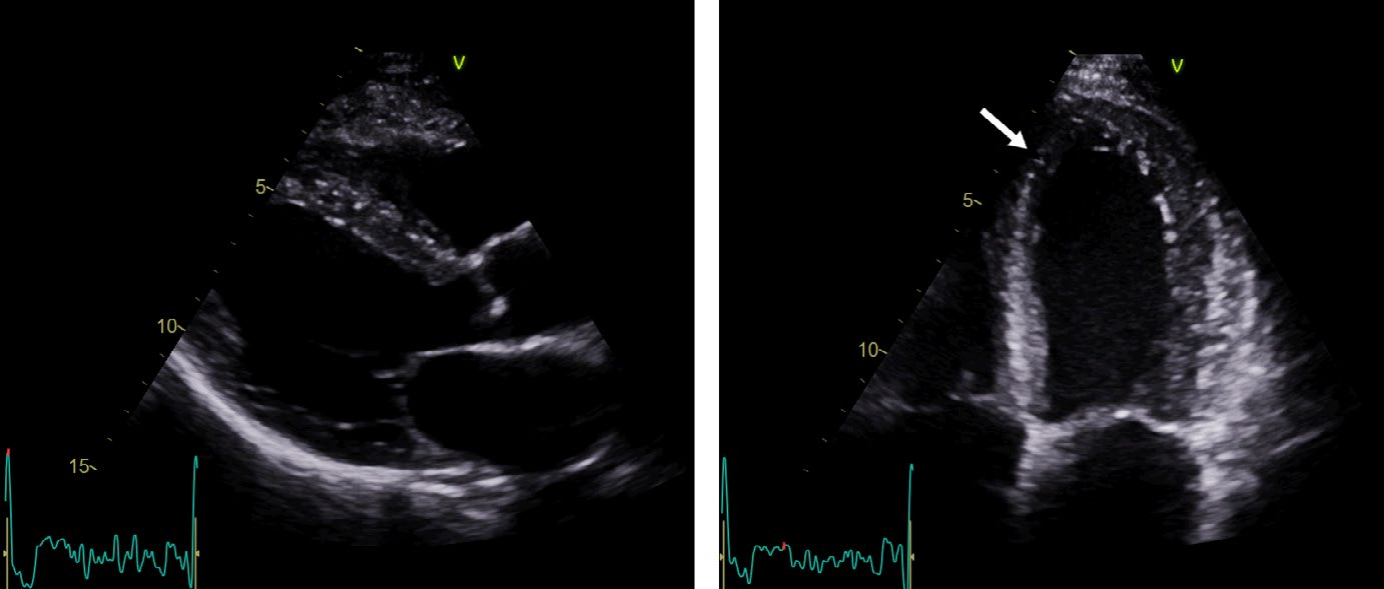
Left hand panel shows a end‐diastolic parasternal long axis echo image. No left ventricular hypertrophy or valve disease was found. Right hand panel shows a end‐systolic apical 4 chamber view with anteroseptal hypokinesia (white arrow)

**Figure 6:**
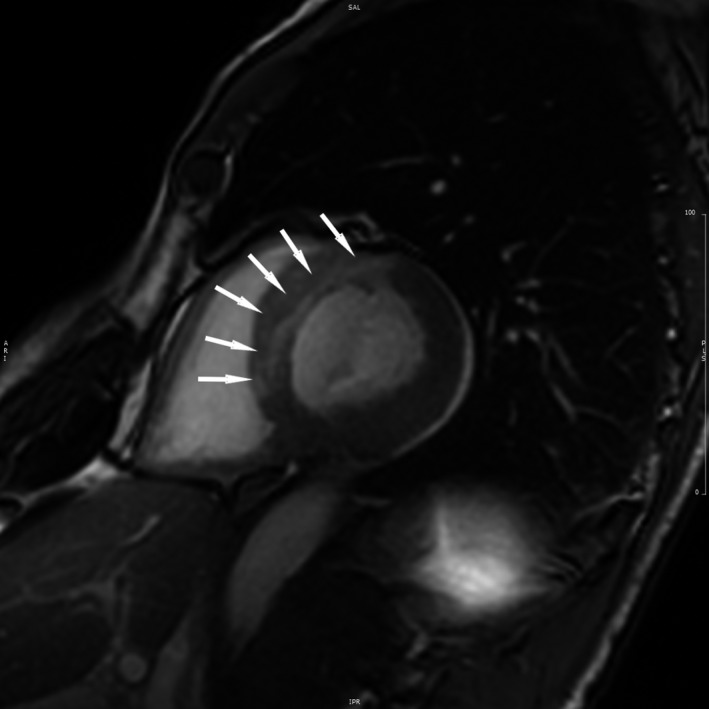
MRI scan showing large anteroseptal myocardial infarction (white arrows)

Following PPCI and resuscitation, the patient was fully awake and aware but with symptoms of slight chest discomfort and nausea. Vital signs were stable, and a new 12‐lead ECG was recorded demonstrating sinus rhythm with resolved ST elevations. Echocardiography was performed showing septo‐apical hypokinesia with reduced left ventricular ejection fraction (LVEF) of 40% but was otherwise normal without signs of left ventricular hypertrophy or valve disease (Figure 5). Initial cardiac troponin T was only mildly elevated at 23 ng/L (reference range 0‐14 ng/L) while plasma potassium, sodium and creatinine levels were within normal limits. Repeat measurement of cardiac troponin T the following day showed significantly elevated levels at 7510 ng/L consistent with AMI. Telemetry during the first 24 hours displayed sinus rhythm with multiple episodes of accelerated idioventricular rhythm and nonsustained ventricular tachycardia that was treated acutely with two doses of metoprolol 50 mg. No further tachyarrhythmias were observed. Cardiac magnetic resonance imaging (MRI) with stress perfusion was performed demonstrating LVEF of 43% and signs of a fresh anterior AMI with edema and late gadolinium enhancement in the anteroseptal and apical parts of the left ventricle. No other areas with infarction or reversible ischemia were found (Figure 6). After 5 days of observation and telemetry in a coronary ward, the patient was discharged.

## 3 FOLLOW‐UP

Following discharge, the patient was implanted with a 1‐chamber implantable cardioverter‐defibrillator (ICD) 12 days after the index event and referred to an outpatient heart failure clinic where he was up‐titrated in anti‐congestive medication. At 6 months from index event, no further sustained tachyarrhythmias have been recorded from the ICD and repeat echocardiography has demonstrated normalization of left ventricular systolic function with LVEF of 55%. The patient is asymptomatic and has resumed participating in orienteering races.

## 4 DISCUSSION

Ventricular tachycardia (VT) and ventricular fibrillation (VF) are important complications in both acute and chronic CAD. Traditionally acute ischemia is thought to give rise to VF, occasionally preceded by polymorphic VT, whereas SMVT is associated with chronic CAD where surviving myocardial cells in the border zone of a healed infarction is able to serve as a substrate for reentry.^1^, ^2^, ^3^, ^4^, ^5^

In this report, we present a case of SMVT as the initial presentation of STEMI in a previously healthy individual. This suggests that SMVT can develop and persist in the setting of extensive acute ischemia in the absence of a known pre‐existing substrate. While the possibility of antecedent occult CAD in the patient cannot be ruled out, there are several conditions that reduce the likelihood of this being the case. The patient did not report of any cardiac symptoms prior to the event despite regular strenuous exercise, had a normal ECG from 2 years past, and cardiac MRI with stress perfusion and LGE did not reveal other areas with ischemia or previous infarction.

Based on the surface ECGs, the exact nature of the SMVT cannot be established. A possible mechanism could be macro‐reentry with the large ischemic parts of the ventricle containing areas of slow conduction and block mimicking the electrophysiological properties of myocardial scar. Other possible mechanisms could be focal such as increased automaticity combined with the mechanical stretching of the ischemic areas of the left ventricle, and a high sympathetic tone that participation in the orienteering race might also have contributed to. At admittance to hospital, the plasma potassium levels of the patient were within normal limits, but had hypokalemia been present this could have increased the likelihood of triggering and sustaining the SMVT.

The observation that SMVT can occur in previously healthy individuals in the setting of acute ischemia is in line with a study from Hatzinikolaou‐Kotsakou et al^6^ where SMVT was found in 0.6% of patients within the first 6 hours of AMI. This incidence is echoed in a similar study from Mont et al^7^ where 0.8% of patients developed SMVT within the first 4 hours of AMI; however, this study did not exclude patients with previous CAD. In both studies, patients were treated with thrombolysis and the occurrence of SMVT was found associated with extensive infarction size, predominantly anterior of origin as the case in our patient. In a more recent study by Hai et al^8^ where patients treated with PCI were included, SMVT was found in 3.3% of the study population however this incidence was within the first 2 days of AMI rather than within the first hours. Only few studies examining the incidence of ventricular tachyarrhythmias in STEMI before PPCI as in the present case report exist.^9^, ^10^ These suggest a VT/VF incidence of 0.4%‐3.0% but do not differentiate between SMVT and VF let alone polymorphic VT.

## 5 CONCLUSION

In rare cases, sustained monomorphic VT can be the initial presentation of an AMI in previously healthy individuals.

## CONFLICT OF INTEREST

None declared.

## AUTHOR CONTRIBUTIONS

JAA: treating physician, drafting of the initial and revised manuscript. Corresponding author. PH: contributed and consulted in parts regarding interventional cardiology and cardiac imaging. JML: contributed and consulted in parts regarding electrophysiology. NHA: idea and conceptualization. All authors approved the final version of the case report for submission to the Clinical Case Reports.
